# Supplementary material for: Theoretical Study on the Open-Shell Electronic Structure and Electron Conductivity of [18]Annulene as a Molecular Parallel Circuit Model
Source: Nanomaterials (Basel). 2023 Dec 31;14(1):98. doi: 10.3390/nano14010098 (PMC10781064; doi:10.3390/nano14010098)
Supplement: Supplementary file 1 [file nanomaterials-14-00098-s001.zip › nanomaterials-2705484-supplementary.pdf]

Supplementary Materials for

Theoretical Study on the Open-Shell Electronic Structure and Electron Conductivity of [18]Annulene as a Molecular Parallel Circuit Model

Naoka Amamizu [1,*], Mitsuhiro Nishida [1], Keisuke Sasaki [1], Ryohei Kishi [1,2,3,4] and Yasutaka Kitagawa [1,2,3,4,5,*]

[1] Department of Materials Engineering Science, Graduate School of Engineering Science, Osaka University, Toyonaka, Osaka 560-8531, Japan; mitsuhiro.nishida@cheng.es.osaka-u.ac.jp (M.N.); keisuke.sasaki@cheng.es.osaka-u.ac.jp (K.S.); kishi.ryohei.es@osaka-u.ac.jp (R.K.)
[2] Center for Quantum Information and Quantum Biology (QIQB), International Advanced Research Institute (IARI), Osaka University, Toyonaka, Osaka 560-0043, Japan
[3] Research Center for Solar Energy Chemistry (RCSEC), Graduate School of Engineering Science, Osaka University, Toyonaka, Osaka 560-8531, Japan
[4] Innovative Catalysis Science Division, Institute for Open and Transdisciplinary Research Initiatives (ICS-OTRI), Osaka University, Suita, Osaka 565-0871, Japan
[5] Spintronics Research Network Division, Institute for Open and Transdisciplinary Research Initiatives (SRN-OTRI), Osaka University, Toyonaka, Osaka 560-8531, Japan
* Correspondence: (N.A.); kitagawa.yasutaka.es@osaka-u.ac.jp (Y.K.)

Table S1. Cartesian coordinate of (a)Non-Substituted(NS), (b)OCH_3-substituted, (c)OH-substituted, (d)CN-substituted and (e)NO_2-substituted molecules.

(a)

	NS						
	Annulene						
atom	x / Å	y / Å	z / Å	atom	x / Å	y / Å	z / Å
C	-1.391967	-2.450151	-0.198955	H	3.275459	3.365302	0.320865
C	-0.574917	-3.631100	-0.089730	C	-3.099538	-0.018012	0.045606
C	0.771962	-3.641858	0.001599	H	-4.454208	-1.270807	-0.928810
C	1.599641	-2.465048	0.040978	C	0.574917	3.631100	0.089730
C	2.944633	-2.464386	0.087475	H	0.882169	1.503045	0.251534
C	3.765525	-1.262846	0.177444	C	-3.765525	1.262846	-0.177444
C	3.099538	0.018012	-0.045606	H	-2.187624	0.015885	0.618128
C	3.506561	1.209227	0.415724	C	-0.771962	3.641858	-0.001599
C	-2.732033	-2.431854	-0.288375	H	1.093392	4.578333	0.093572
H	-0.882169	-1.503045	-0.251534	C	-2.944633	2.464386	-0.087475
H	-1.093392	-4.578333	-0.093572	C	-1.599641	2.465048	-0.040978
H	1.276809	-4.594271	0.055359	H	-1.276809	4.594271	-0.055359
H	1.086995	-1.520549	0.057602	H	-3.482423	3.398725	-0.112476
H	3.482423	-3.398725	0.112476	H	-1.086995	1.520549	-0.057602
H	2.187624	-0.015885	-0.618128	S	-5.369939	1.400613	-0.528373
H	4.454208	1.270807	0.928810	S	5.369939	-1.400613	0.528373
H	-3.275459	-3.365302	-0.320865	Au	-7.470426	3.024416	-1.089461
C	2.732033	2.431854	0.288375	Au	-7.747486	0.161328	-0.946753
C	-3.506561	-1.209227	-0.415724	Au	7.470426	-3.024416	1.089461
C	1.391967	2.450151	0.198955	Au	7.747486	-0.161328	0.946753

atom	Polyene A			atom	Polyene B		
	x / Å	y / Å	z / Å		x / Å	y / Å	z / Å
C	3.765526	-1.262846	0.177444	C	-1.391967	-2.450151	-0.198955
C	3.099538	0.018012	-0.045606	C	-0.574917	-3.631101	-0.089730
C	3.506562	1.209227	0.415724	C	0.771962	-3.641859	0.001599
H	2.187624	-0.015885	-0.618128	C	1.599641	-2.465048	0.040978

H	4.454209	1.270807	0.928810	C	2.944633	-2.464386	0.087475
C	2.732033	2.431854	0.288375	C	3.765526	-1.262846	0.177444
C	1.391967	2.450151	0.198955	C	-2.732033	-2.431854	-0.288375
H	3.275460	3.365303	0.320865	H	-0.882169	-1.503045	-0.251534
C	0.574917	3.631101	0.089730	H	-1.093392	-4.578334	-0.093572
H	0.882169	1.503045	0.251534	H	1.276809	-4.594272	0.055359
C	-3.765526	1.262846	-0.177444	H	1.086995	-1.520549	0.057602
C	-0.771962	3.641859	-0.001599	H	3.482424	-3.398726	0.112476
H	1.093392	4.578334	0.093572	H	-3.275460	-3.365303	-0.320865
C	-2.944633	2.464386	-0.087475	C	-3.506562	-1.209227	-0.415724
C	-1.599641	2.465048	-0.040978	C	-3.099538	-0.018012	0.045606
H	-1.276809	4.594272	-0.055359	H	-4.454209	-1.270807	-0.928810
H	-3.482424	3.398726	-0.112476	C	-3.765526	1.262846	-0.177444
H	-1.086995	1.520549	-0.057602	H	-2.187624	0.015885	0.618128
S	-5.369940	1.400613	-0.528373	S	-5.369940	1.400613	-0.528373
S	5.369940	-1.400613	0.528373	S	5.369940	-1.400613	0.528373
H	-3.285134	0.300341	-0.057855	H	-3.158309	2.147995	-0.042461
H	3.158309	-2.147995	0.042461	H	3.285134	-0.300341	0.057855
Au	-7.470427	3.024416	-1.089461	Au	-7.470427	3.024416	-1.089461
Au	-7.747487	0.161328	-0.946753	Au	-7.747487	0.161328	-0.946753
Au	7.470427	-3.024416	1.089461	Au	7.470427	-3.024416	1.089461
Au	7.747487	-0.161328	0.946753	Au	7.747487	-0.161328	0.946753

(b)

	OCH3						
	Annulene						
atom	x / Å	y / Å	z / Å	atom	x / Å	y / Å	z / Å
C	-1.361122	-2.450887	-0.153892	H	-2.228204	-0.037362	0.781358
C	-0.522548	-3.604530	-0.023031	C	-0.833338	3.603853	-0.024081
C	0.831135	-3.602221	0.021132	H	1.027381	4.555207	-0.045684
C	1.643341	-2.423677	-0.018904	C	-2.995682	2.407839	-0.029550
C	2.993479	-2.406206	0.026610	C	-1.645544	2.425309	0.015963

C	3.798792	-1.205229	0.057027	H	-1.345998	4.549161	-0.113009
C	3.116484	0.073064	-0.178368	H	-3.538374	3.336569	-0.103932
C	3.498845	1.253357	0.316785	H	-1.121577	1.486407	0.056962
C	-2.712893	-2.472778	-0.175202	S	-5.422943	1.295512	-0.354698
H	-0.904428	-1.489286	-0.293190	S	5.420742	-1.293883	0.351748
H	-1.029584	-4.553575	0.042727	O	3.341269	3.662470	0.138415
H	1.343795	-4.547530	0.110054	O	-3.343471	-3.660836	-0.141379
H	1.119374	-1.484774	-0.059895	C	4.713246	3.714421	-0.196416
H	3.536171	-3.334937	0.100982	H	4.922626	4.750273	-0.431130
H	2.225998	0.038991	-0.784286	H	5.337196	3.407778	0.638877
H	4.419161	1.309838	0.876090	H	4.934551	3.094017	-1.060535
C	2.710690	2.474412	0.172259	C	-4.715454	-3.712791	0.193427
C	-3.501045	-1.251721	-0.319728	H	-5.339390	-3.406140	-0.641873
C	1.358920	2.452520	0.150950	H	-4.924838	-4.748645	0.428126
C	-3.118688	-0.071432	0.175436	H	-4.936774	-3.092396	1.057548
H	-4.421358	-1.308198	-0.879039	Au	-7.583310	2.850914	-0.881138
C	0.520345	3.606162	0.020082	Au	-7.781924	-0.012878	-0.649527
H	0.902226	1.490920	0.290254	Au	7.581118	-2.849293	0.878127
C	-3.800996	1.206862	-0.059953	Au	7.779725	0.014504	0.646572

atom	Polyene A			atom	Polyene B		
	x / Å	y / Å	z / Å		x / Å	y / Å	z / Å
C	3.798844	-1.205143	0.057049	C	-1.361122	-2.450887	-0.153892
C	3.116439	0.073035	-0.178391	C	-0.522548	-3.60453	-0.023031
C	3.498839	1.253355	0.316794	C	0.831135	-3.602221	0.021132
H	2.225999	0.038991	-0.784288	C	1.643341	-2.423677	-0.018904
H	4.419161	1.309838	0.876090	C	2.993472	-2.406201	0.026609
C	2.710690	2.474412	0.172259	C	3.798799	-1.205242	0.057028
C	1.358920	2.452520	0.150950	C	-2.712893	-2.472778	-0.175202
C	0.520345	3.606162	0.020082	H	-0.904428	-1.489286	-0.29319
H	0.902226	1.490920	0.290254	H	-1.029584	-4.553575	0.042727
C	-3.801003	1.206875	-0.059954	H	1.343795	-4.54753	0.110054
C	-0.833338	3.603853	-0.024081	H	1.119374	-1.484774	-0.059895

H	1.027381	4.555207	-0.045684	H	3.536171	-3.334937	0.100981
C	-2.995675	2.407834	-0.029549	C	-3.501039	-1.251719	-0.319737
C	-1.645544	2.425309	0.015963	C	-3.118643	-0.071403	0.175459
H	-1.345998	4.549161	-0.113009	H	-4.421358	-1.308198	-0.879039
H	-3.538374	3.336569	-0.103931	C	-3.801048	1.206776	-0.059975
H	-1.121577	1.486407	0.056962	H	-2.228205	-0.037362	0.781360
S	-5.422943	1.295504	-0.354698	S	-5.422941	1.295568	-0.354692
S	5.420740	-1.293939	0.351742	S	5.420742	-1.293875	0.351748
O	3.341269	3.662470	0.138415	O	-3.343471	-3.660836	-0.141379
C	4.713246	3.714421	-0.196416	C	-4.715454	-3.712791	0.193427
H	4.922626	4.750273	-0.431130	H	-5.339390	-3.406140	-0.641873
H	5.337196	3.407778	0.638877	H	-4.924838	-4.748645	0.428126
H	4.934551	3.094017	-1.060535	H	-4.936774	-3.092396	1.057548
H	-3.303408	0.254955	0.076925	H	-3.205474	2.104015	0.037887
H	3.203265	-2.102381	-0.040795	H	3.301201	-0.253320	-0.079828
Au	-7.58331	2.850914	-0.881138	Au	-7.583310	2.850914	-0.881138
Au	-7.781924	-0.012878	-0.649527	Au	-7.781924	-0.012878	-0.649527
Au	7.581118	-2.849293	0.878127	Au	7.581118	-2.849293	0.878127
Au	7.779725	0.014504	0.646572	Au	7.779725	0.014504	0.646572

(c)

	OH						
	Annulene						
atom	x / Å	y / Å	z / Å	atom	x / Å	y / Å	z / Å
C	-1.355180	-2.439524	-0.197345	C	0.539428	3.610492	0.062134
C	-0.539789	-3.610226	-0.062652	H	0.878771	1.482067	0.289339
C	0.811841	-3.627818	0.019507	C	-3.793015	1.246817	-0.139081
C	1.636382	-2.456700	0.017092	H	-2.226359	-0.004835	0.701312
C	2.985586	-2.446090	0.083444	C	-0.812203	3.628085	-0.020011
C	3.792647	-1.246552	0.138676	H	1.062175	4.552985	0.029934
C	3.111264	0.034361	-0.088218	C	-2.985949	2.446355	-0.083896
C	3.480794	1.211386	0.422326	C	-1.636745	2.456967	-0.017560

C	-2.701524	-2.434808	-0.274908	H	-1.310884	4.581046	-0.104292
H	-0.879132	-1.481796	-0.289804	H	-3.525591	3.377249	-0.152193
H	-1.062535	-4.552721	-0.030482	H	-1.120116	1.514204	0.016178
H	1.310524	-4.580781	0.103771	S	-5.410685	1.336475	-0.449602
H	1.119754	-1.513936	-0.016626	S	5.410331	-1.336222	0.449127
H	3.525229	-3.376985	0.151716	O	3.377781	3.603160	0.290856
H	2.225998	0.005084	-0.701755	H	4.316592	3.448564	0.179318
H	4.394308	1.275009	0.996903	O	-3.378144	-3.602888	-0.291380
C	2.701164	2.435079	0.274403	H	-4.316956	-3.448292	-0.179850
C	-3.481151	-1.211110	-0.422804	Au	-7.568134	2.894897	-0.979069
C	1.354819	2.439793	0.196850	Au	-7.764083	0.028063	-0.786018
C	-3.111623	-0.034099	0.087774	Au	7.567809	-2.894667	0.978406
H	-4.394663	-1.274717	-0.997387	Au	7.763736	-0.027820	0.785535

atom	Polyene A			atom	Polyene B		
	x / Å	y / Å	z / Å		x / Å	y / Å	z / Å
C	3.792698	-1.246470	0.138699	C	-1.355180	-2.439524	-0.197345
C	3.111221	0.034334	-0.088241	C	-0.539789	-3.610227	-0.062652
C	3.480789	1.211384	0.422334	C	0.811841	-3.627819	0.019507
H	2.226000	0.005084	-0.701757	C	1.636382	-2.456700	0.017092
H	4.394309	1.275009	0.996903	C	2.985579	-2.446085	0.083443
C	2.701164	2.435079	0.274403	C	3.792655	-1.246565	0.138677
C	1.354819	2.439793	0.196850	C	-2.701524	-2.434808	-0.274908
C	0.539428	3.610493	0.062134	H	-0.879132	-1.481796	-0.289804
H	0.878771	1.482067	0.289339	H	-1.062535	-4.552722	-0.030482
C	-3.793022	1.246830	-0.139082	H	1.310524	-4.580782	0.103771
C	-0.812203	3.628086	-0.020011	H	1.119754	-1.513936	-0.016626
H	1.062175	4.552986	0.029934	H	3.525230	-3.376986	0.151715
C	-2.985942	2.446350	-0.083895	C	-3.481146	-1.211108	-0.422812
C	-1.636745	2.456967	-0.017560	C	-3.111580	-0.034072	0.087797
H	-1.310884	4.581047	-0.104292	H	-4.394664	-1.274717	-0.997387
H	-3.525592	3.377250	-0.152192	C	-3.793066	1.246734	-0.139104
H	-1.120116	1.514204	0.016178	H	-2.226361	-0.004835	0.701314

S	-5.410686	1.336467	-0.449602	S	-5.410684	1.336529	-0.449596
S	5.410330	-1.336276	0.449121	S	5.410332	-1.336214	0.449127
O	3.377782	3.603161	0.290856	O	-3.378145	-3.602889	-0.291380
H	4.316593	3.448565	0.179318	H	-4.316957	-3.448293	-0.179850
H	-3.296341	0.293471	-0.008361	H	-3.198241	2.142567	-0.025689
H	3.197856	-2.142300	0.025350	H	3.295965	-0.293201	0.008027
Au	-7.568154	2.894862	-0.979076	Au	-7.588904	2.899593	-0.868870
Au	-7.764069	0.028026	-0.786012	Au	-7.743300	0.023864	-0.896151
Au	7.588544	-2.899356	0.868363	Au	7.567829	-2.894632	0.978412
Au	7.74298739	-0.023628	0.8955125	Au	7.763722	-0.027783	0.785530

(d)

	CN						
	Annulene						
atom	x / Å	y / Å	z / Å	atom	x / Å	y / Å	z / Å
C	-1.398425	-2.500924	-0.249187	C	0.563779	3.663326	0.141204
C	-0.563779	-3.663326	-0.141204	H	0.910580	1.543657	0.292506
C	0.782990	-3.628616	-0.039198	C	-3.752043	1.229477	-0.204090
C	1.586414	-2.435816	0.020347	H	-2.185630	-0.037074	0.591276
C	2.929787	-2.429626	0.087330	C	-0.782990	3.628616	0.039198
C	3.752043	-1.229477	0.204090	H	1.054659	4.623532	0.154411
C	3.091003	0.058995	-0.007718	C	-2.929787	2.429626	-0.087330
C	3.510617	1.235349	0.471866	C	-1.586414	2.435816	-0.020347
C	-2.747012	-2.476195	-0.349772	H	-1.311751	4.568031	-0.012097
H	-0.910580	-1.543657	-0.292506	H	-3.468871	3.363241	-0.108748
H	-1.054659	-4.623532	-0.154411	H	-1.063462	1.496610	-0.037256
H	1.311751	-4.568031	0.012097	S	-5.346406	1.376991	-0.572012
H	1.063462	-1.496610	0.037256	S	5.346406	-1.376991	0.572012
H	3.468871	-3.363241	0.108748	C	3.485828	3.702566	0.401066
H	2.185630	0.037074	-0.591276	N	4.079994	4.684694	0.443640
H	4.453430	1.288364	0.993158	C	-3.485828	-3.702566	-0.401066
C	2.747012	2.476195	0.349772	N	-4.079994	-4.684694	-0.443640

C	-3.510617	-1.235349	-0.471866	Au	-7.434559	3.015444	-1.136537
C	1.398425	2.500924	0.249187	Au	-7.722353	0.151566	-1.037642
C	-3.091003	-0.058995	0.007718	Au	7.434559	-3.015444	1.136537
H	-4.453430	-1.288364	-0.993158	Au	7.722353	-0.151566	1.037642

atom	Polyene A			atom	Polyene B		
	x / Å	y / Å	z / Å		x / Å	y / Å	z / Å
C	3.752044	-1.229477	0.204090	C	-1.398425	-2.500924	-0.249187
C	3.091003	0.058995	-0.007718	C	-0.563779	-3.663327	-0.141204
C	3.510618	1.235349	0.471866	C	0.782990	-3.628617	-0.039198
H	2.185630	0.037074	-0.591276	C	1.586414	-2.435816	0.020347
H	4.453431	1.288364	0.993158	C	2.929787	-2.429626	0.087330
C	2.747012	2.476195	0.349772	C	3.752044	-1.229477	0.204090
C	1.398425	2.500924	0.249187	C	-2.747012	-2.476195	-0.349772
C	0.563779	3.663327	0.141204	H	-0.910580	-1.543657	-0.292506
H	0.910580	1.543657	0.292506	H	-1.054659	-4.623533	-0.154411
C	-3.752044	1.229477	-0.204090	H	1.311751	-4.568032	0.012097
C	-0.782990	3.628617	0.039198	H	1.063462	-1.496610	0.037256
H	1.054659	4.623533	0.154411	H	3.468872	-3.363242	0.108748
C	-2.929787	2.429626	-0.087330	C	-3.510618	-1.235349	-0.471866
C	-1.586414	2.435816	-0.020347	C	-3.091003	-0.058995	0.007718
H	-1.311751	4.568032	-0.012097	H	-4.453431	-1.288364	-0.993158
H	-3.468872	3.363242	-0.108748	C	-3.752044	1.229477	-0.204090
H	-1.063462	1.496610	-0.037256	H	-2.185630	-0.037074	0.591276
S	-5.346407	1.376991	-0.572012	S	-5.346407	1.376991	-0.572012
S	5.346407	-1.376991	0.572012	S	5.346407	-1.376991	0.572012
C	3.485829	3.702567	0.401066	C	-3.485829	-3.702567	-0.401066
N	4.079995	4.684695	0.443640	N	-4.079995	-4.684695	-0.443640
H	-3.274920	0.264701	-0.089865	H	-3.141303	2.108928	-0.049166
H	3.141303	-2.108928	0.049166	H	3.274920	-0.264701	0.089865
Au	-7.434560	3.015444	-1.136537	Au	-7.457218	3.017536	-1.037514
Au	-7.722354	0.151567	-1.037642	Au	-7.699696	0.149475	-1.136665
Au	7.457218	-3.017536	1.037514	Au	7.434560	-3.015444	1.136537

Au	7.699696	-0.149475	1.136665	Au	7.722354	-0.151567	1.037642

(e)

	NO2						
	Annulene						
atom	x / Å	y / Å	z / Å	atom	x / Å	y / Å	z / Å
C	-1.446571	-2.655699	-0.303308	H	0.975926	1.689960	0.357848
C	-0.547593	-3.768546	-0.194653	C	-3.689923	1.157192	-0.259277
C	0.793238	-3.622814	-0.084483	H	-2.123598	-0.119624	0.520428
C	1.535501	-2.392248	-0.006385	C	-0.793235	3.622816	0.084427
C	2.875782	-2.359417	0.099877	H	0.957929	4.758539	0.209772
C	3.689923	-1.157195	0.259267	C	-2.875778	2.359415	-0.099899
C	3.041717	0.135931	0.043692	C	-1.535494	2.392248	0.006331
C	3.510741	1.306899	0.491080	H	-1.382313	4.525907	0.029547
C	-2.793026	-2.567993	-0.373859	H	-3.424348	3.287295	-0.127604
H	-0.975928	-1.689945	-0.358012	H	-0.982931	1.469393	0.000443
H	-0.957938	-4.758522	-0.209826	S	-5.267540	1.325516	-0.682931
H	1.382309	-4.525908	-0.029593	S	5.267572	-1.325524	0.682800
H	0.982941	-1.469391	-0.000514	N	3.645920	3.749798	0.387513
H	3.424352	-3.287297	0.127590	O	3.128369	4.846824	0.413966
H	2.123610	0.119615	-0.520466	O	4.842563	3.562665	0.367055
H	4.468231	1.335634	0.981699	N	-3.645916	-3.749800	-0.387317
C	2.793026	2.567995	0.373893	O	-4.842558	-3.562666	-0.366599
C	-3.510763	-1.306909	-0.491054	O	-3.128378	-4.846831	-0.413925
C	1.446575	2.655717	0.303237	Au	-7.318061	2.992257	-1.300134
C	-3.041719	-0.135935	-0.043706	Au	-7.636221	0.130282	-1.252462
H	-4.468267	-1.335661	-0.981646	Au	7.318157	-2.992275	1.299762
C	0.547595	3.768560	0.194594	Au	7.636278	-0.130294	1.252233

atom	Polyene A			atom	Polyene B		
	x / Å	y / Å	z / Å		x / Å	y / Å	z / Å
C	3.689923	-1.157195	0.259267	C	-1.446571	-2.655699	-0.303308
C	3.041717	0.135931	0.043692	C	-0.547593	-3.768546	-0.194653

C	3.510741	1.306899	0.491080	C	0.793238	-3.622814	-0.084483
H	2.123610	0.119615	-0.520466	C	1.535501	-2.392248	-0.006385
H	4.468231	1.335634	0.981699	C	2.875782	-2.359417	0.099877
C	2.793026	2.567995	0.373893	C	3.689923	-1.157195	0.259267
C	1.446575	2.655717	0.303237	C	-2.793026	-2.567993	-0.373859
C	0.547595	3.768560	0.194594	H	-0.975928	-1.689945	-0.358012
H	0.975926	1.689960	0.357848	H	-0.957938	-4.758522	-0.209826
C	-3.689923	1.157192	-0.259277	H	1.382309	-4.525908	-0.029593
C	-0.793235	3.622816	0.084427	H	0.982941	-1.469391	-0.000514
H	0.957929	4.758539	0.209772	H	3.424352	-3.287297	0.12759
C	-2.875778	2.359415	-0.099899	C	-3.510763	-1.306909	-0.491054
C	-1.535494	2.392248	0.006331	C	-3.041719	-0.135935	-0.043706
H	-1.382313	4.525907	0.029547	H	-4.468267	-1.335661	-0.981646
H	-3.424348	3.287295	-0.127604	C	-3.689923	1.157192	-0.259277
H	-0.982931	1.469393	0.000443	H	-2.123598	-0.119624	0.520428
S	-5.267540	1.325516	-0.682931	S	-5.267540	1.325516	-0.682931
S	5.267572	-1.325524	0.682800	S	5.267572	-1.325524	0.682800
N	3.645920	3.749798	0.387513	N	-3.645916	-3.749800	-0.387317
O	3.128369	4.846824	0.413966	O	-4.842558	-3.562666	-0.366599
O	4.842563	3.562665	0.367055	O	-3.128378	-4.846831	-0.413925
H	-3.221164	0.189072	-0.141349	H	-3.079082	2.030480	-0.072766
H	3.079058	-2.030482	0.072830	H	3.221150	-0.189075	0.141402
Au	-7.318061	2.992257	-1.300134	Au	-7.318061	2.992257	-1.300134
Au	-7.636221	0.130282	-1.252462	Au	-7.636221	0.130282	-1.252462
Au	7.318157	-2.992275	1.299762	Au	7.318157	-2.992275	1.299762
Au	7.636278	-0.130294	1.252233	Au	7.636278	-0.130294	1.252233

Table S2. Calculated parameters related to the transition probability of (a)NS, (b)OCH_3-substituted, (c)OH-substituted, (d)CN-substituted and (e)NO_2-substituted molecules. (x-1)Site-overlap, (x-2)square of an expansion coefficient of the wave function of the molecule at the end-site, interaction between the LUMO of the molecule and the HOMO of the electrode, HOMO-LUMO gap of extended molecule, Fermi energy of electrode and coupling constants (x: a, b, c, d, e).

The subscripts σ in table(x-2) represents α or β orbital.

(a-1)

	NS					
	Site-overlap					
	Annulene		Polyene A		Polyene B	
	α orbital	β orbital	α orbital	β orbital	α orbital	β orbital
HOMO-9	0.0000	0.0000	0.0002	0.0000	0.0001	0.0000
HOMO-8	0.0213	0.0213	0.0000	0.0002	0.0000	0.0001
HOMO-7	0.0158	0.0158	0.0000	0.0000	0.0000	0.0000
HOMO-6	0.4074	0.4074	0.0000	0.0000	0.0000	0.0000
HOMO-5	2.2823	2.2823	0.2068	0.1903	0.1287	0.1534
HOMO-4	0.9860	0.9860	0.0043	1.5019	0.0036	1.4478
HOMO-3	0.0033	0.0033	0.0557	0.9201	0.0465	0.8951
HOMO-2	0.0150	0.0150	0.0015	0.0035	0.0000	0.0023
HOMO-1	0.0321	0.0321	0.0000	0.0000	0.0000	0.0000
HOMO	0.0331	0.0331	0.0000	0.0000	0.0000	0.0000
LUMO	0.0703	0.0703	0.0578	0.0351	0.0453	0.0515
LUMO+1	0.0341	0.0341	0.0002	0.0001	0.0000	0.0000
LUMO+2	0.0001	0.0001	0.0099	0.0013	0.0022	0.0001
LUMO+3	0.0023	0.0023	0.0308	0.0323	0.0342	0.0593
LUMO+4	0.0003	0.0003	0.0151	0.0112	0.0127	0.0158
LUMO+5	0.0037	0.0037	0.0000	0.0000	0.0030	0.0027
LUMO+6	0.0130	0.0130	0.0003	0.0002	0.0000	0.0000
LUMO+7	0.0000	0.0000	0.0135	0.0128	0.0310	0.0294
LUMO+8	0.0001	0.0001	0.0016	0.0016	0.0018	0.0017
LUMO+9	0.0170	0.0170	0.0000	0.0000	0.0000	0.0000
Total	3.9372	3.9372	0.3976	2.7105	0.3092	2.6593

(a-2)

	NS					
	Annulene		Polyene A		Polyene B	
	α orbital	β orbital	α orbital	β orbital	α orbital	β orbital
${d_{1,\sigma}}^2$	0.140	0.140	0.231	0.089	0.090	0.217
${d_{N,\sigma}}^2$	0.140	0.140	0.090	0.217	0.231	0.089
${V_{L,\sigma}}^2$ / eV^2	5.22	5.22	5.04	5.08	5.04	5.08
HOMO-LUMO gap / eV	2.67	2.67	2.93	2.93	2.93	2.93
Fermi energy / eV	-4.59	-4.59	-4.55	-4.55	-4.55	-4.55
$\gamma_{L1,\sigma}$ / eV	0.855	0.855	1.078	0.674	0.675	1.049
$\gamma_{NR,\sigma}$ / eV	0.855	0.855	0.675	1.049	1.078	0.674
${\gamma_{L1,\sigma}}^2{\gamma_{NR,\sigma}}^2$ / eV^4	0.534	0.534	0.530	0.500	0.530	0.500

(b-1)

	OCH_3					
	Site-overlap					
	Annulene		Polyene A		Polyene B	
	α orbital	β orbital	α orbital	β orbital	α orbital	β orbital
HOMO-9	0.0000	0.0000	0.0000	0.0000	0.0000	0.0000
HOMO-8	0.0171	0.0171	0.0000	0.0000	0.0000	0.0000
HOMO-7	0.0079	0.0079	0.0000	0.0000	0.0000	0.0000
HOMO-6	0.4982	0.4982	0.0002	0.0001	0.0001	0.0002
HOMO-5	1.2606	1.2606	0.1801	0.1947	0.1125	0.1317
HOMO-4	0.9051	0.9051	0.0005	0.0001	0.0001	0.0005
HOMO-3	0.1147	0.1147	0.0036	0.0031	0.0024	0.0033
HOMO-2	0.0000	0.0000	0.0312	0.0521	0.0291	0.0260
HOMO-1	0.0313	0.0313	0.0003	0.0004	0.0001	0.0001
HOMO	0.0369	0.0369	0.0000	0.0000	0.0000	0.0000
LUMO	0.0968	0.0968	0.0334	0.0960	0.0688	0.0448
LUMO+1	0.0043	0.0043	0.0001	0.0003	0.0001	0.0000
LUMO+2	0.0000	0.0000	0.0253	0.1044	0.0673	0.0270

LUMO+3	0.0373	0.0373	0.0017	0.0001	0.0002	0.0044
LUMO+4	0.0003	0.0003	0.0119	0.0181	0.0183	0.0195
LUMO+5	0.0022	0.0022	0.0000	0.0000	0.0009	0.0006
LUMO+6	0.0238	0.0238	0.0035	0.0046	0.0154	0.0127
LUMO+7	0.0001	0.0001	0.0000	0.0000	0.0000	0.0000
LUMO+8	0.0000	0.0000	0.0001	0.0003	0.0054	0.0052
LUMO+9	0.0173	0.0173	0.0006	0.0005	0.0036	0.0038
Total	3.0539	3.0539	0.2926	0.4748	0.3243	0.2798

(b-2)

	OCH_3					
	Annulene		Polyene A		Polyene B	
	α orbital	β orbital	α orbital	β orbital	α orbital	β orbital
${d_{1,\sigma}}^2$	0.135	0.135	0.040	0.135	0.169	0.293
${d_{N,\sigma}}^2$	0.135	0.135	0.293	0.169	0.135	0.040
${V_{L,\sigma}}^2$ / eV^2	5.30	5.30	5.61	5.73	5.73	5.61
HOMO-LUMO gap / eV	2.79	2.79	2.76	2.76	2.76	2.76
Fermi energy / eV	-4.52	-4.52	-4.43	-4.43	-4.43	-4.43
$\gamma_{L1,\sigma}$ / eV	0.847	0.847	0.474	0.880	0.983	1.283
$\gamma_{NR,\sigma}$ / eV	0.847	0.847	1.283	0.983	0.880	0.474
${\gamma_{L1,\sigma}}^2{\gamma_{NR,\sigma}}^2$ / eV^4	0.514	0.514	0.370	0.748	0.748	0.370

(c-1)

	OH					
	Site-overlap					
	Annulene		Polyene A		Polyene B	
	α orbital	β orbital	α orbital	β orbital	α orbital	β orbital
HOMO-9	0.0000	0.0000	0.0000	0.0000	0.0000	0.0000
HOMO-8	0.0187	0.0187	0.0000	0.0000	0.0000	0.0000
HOMO-7	0.0108	0.0108	0.0000	0.0000	0.0000	0.0000
HOMO-6	0.4182	0.4182	0.0700	0.0003	0.0437	0.0003
HOMO-5	1.3370	1.3370	0.0081	0.1576	0.0076	0.0889

HOMO-4	0.9560	0.9560	0.0003	0.0000	0.0002	0.0000
HOMO-3	0.0934	0.0934	0.0048	0.0039	0.0042	0.0031
HOMO-2	0.0001	0.0001	0.0230	0.0420	0.0187	0.0228
HOMO-1	0.0337	0.0337	0.0002	0.0003	0.0000	0.0000
HOMO	0.0383	0.0383	0.0000	0.0000	0.0000	0.0000
LUMO	0.0853	0.0853	0.0278	0.0811	0.0358	0.0559
LUMO+1	0.0271	0.0271	0.0000	0.0000	0.0000	0.0000
LUMO+2	0.0001	0.0001	0.0246	0.0796	0.0290	0.0522
LUMO+3	0.0076	0.0076	0.0003	0.0000	0.0010	0.0001
LUMO+4	0.0001	0.0001	0.0097	0.0149	0.0122	0.0111
LUMO+5	0.0004	0.0004	0.0001	0.0001	0.0063	0.0077
LUMO+6	0.0253	0.0253	0.0031	0.0038	0.0136	0.0149
LUMO+7	0.0004	0.0004	0.0000	0.0000	0.0000	0.0000
LUMO+8	0.0002	0.0002	0.0017	0.0013	0.0266	0.0278
LUMO+9	0.0220	0.0220	0.0005	0.0007	0.0049	0.0029
Total	3.0748	3.0748	0.1740	0.3857	0.2037	0.2878

(c-2)

	OH					
	Annulene		Polyene A		Polyene B	
	α orbital	β orbital	α orbital	β orbital	α orbital	β orbital
${d_{1,\sigma}}^2$	0.137	0.137	0.037	0.123	0.304	0.180
${d_{N,\sigma}}^2$	0.137	0.137	0.304	0.180	0.037	0.123
${V_{L,\sigma}}^2$ / eV^2	5.05	5.05	5.44	5.57	5.44	5.57
HOMO-LUMO gap / eV	2.76	2.76	2.72	2.72	2.72	2.72
Fermi energy / eV	-4.59	-4.59	-4.47	-4.47	-4.47	-4.47
$\gamma_{L1,\sigma}$ / eV	0.832	0.832	0.448	0.828	1.287	1.001
$\gamma_{NR,\sigma}$ / eV	0.832	0.832	1.287	1.001	0.448	0.828
${\gamma_{L1,\sigma}}^2{\gamma_{NR,\sigma}}^2$ / eV^4	0.479	0.479	0.333	0.687	0.333	0.687

(d-1)

	CN					
	Site-overlap					
	Annulene		Polyene A		Polyene B	
	α orbital	β orbital	α orbital	β orbital	α orbital	β orbital
HOMO-9	0.0000	0.0000	0.0000	0.0000	0.0000	0.0000
HOMO-8	0.0016	0.0017	0.0014	0.0000	0.0000	0.0006
HOMO-7	0.0002	0.0002	0.0000	0.0017	0.0010	0.0000
HOMO-6	0.1626	0.1983	0.0000	0.0000	0.0000	0.0000
HOMO-5	0.0006	0.0012	0.0907	0.0786	0.0680	0.0555
HOMO-4	1.5739	1.7597	0.0700	0.3793	0.3695	0.0756
HOMO-3	0.4671	0.4871	0.8267	0.3163	0.3308	0.7286
HOMO-2	0.0205	0.0205	0.1354	0.0440	0.0476	0.1298
HOMO-1	0.0003	0.0003	0.0000	0.0008	0.0009	0.0000
HOMO	0.0005	0.0005	0.0000	0.0012	0.0009	0.0000
LUMO	0.0345	0.0563	0.0415	0.0232	0.0367	0.0308
LUMO+1	0.0196	0.0352	0.0571	0.0308	0.0409	0.0333
LUMO+2	0.0000	0.0000	0.0001	0.0000	0.0000	0.0000
LUMO+3	0.0000	0.0000	0.0003	0.0002	0.0011	0.0007
LUMO+4	0.0027	0.0040	0.0111	0.0077	0.0107	0.0066
LUMO+5	0.0028	0.0057	0.0000	0.0000	0.0000	0.0000
LUMO+6	0.0024	0.0021	0.0001	0.0001	0.0273	0.0200
LUMO+7	0.0000	0.0000	0.0085	0.0065	0.0607	0.0553
LUMO+8	0.0004	0.0001	0.0022	0.0018	0.0023	0.0038
LUMO+9	0.0239	0.0187	0.0000	0.0000	0.0003	0.0002
Total	2.3137	2.5918	1.2451	0.8920	0.9988	1.1409

(d-2)

	CN					
	Annulene		Polyene A		Polyene B	
	α orbital	β orbital	α orbital	β orbital	α orbital	β orbital
${d_{1,\sigma}}^2$	0.063	0.250	0.256	0.085	0.195	0.067
${d_{N,\sigma}}^2$	0.250	0.063	0.068	0.195	0.085	0.256
${V_{L,\sigma}}^2$ / eV^2	3.92	3.92	3.96	4.25	4.25	3.96
HOMO-LUMO gap / eV	2.41	2.41	2.76	2.76	2.76	2.76
Fermi energy / eV	-4.98	-4.98	-4.81	-4.81	-4.81	-4.81
$\gamma_{L1,\sigma}$ / eV	0.497	0.991	1.007	0.602	0.911	0.517
$\gamma_{NR,\sigma}$ / eV	0.991	0.497	0.518	0.911	0.602	1.007
${\gamma_{L1,\sigma}}^2{\gamma_{NR,\sigma}}^2$ / eV^4	0.242	0.242	0.271	0.301	0.301	0.271

(e-1)

	NO_2					
	Site-overlap					
	Annulene		Polyene A		Polyene B	
	α orbital	β orbital	α orbital	β orbital	α orbital	β orbital
HOMO-9	0.0001	0.0001	0.0000	0.0000	0.0000	0.0000
HOMO-8	0.0004	0.0003	0.0000	0.0000	0.0000	0.0000
HOMO-7	0.0001	0.0001	0.0000	0.0000	0.0000	0.0000
HOMO-6	0.1225	0.0987	0.0031	0.0026	0.0017	0.0017
HOMO-5	0.0000	0.0001	0.0538	0.0534	0.0508	0.0367
HOMO-4	0.7107	0.6268	0.2193	0.3221	0.3293	0.1718
HOMO-3	0.1227	0.1153	0.0390	0.0956	0.1047	0.0370
HOMO-2	0.0094	0.0089	0.0000	0.0000	0.0000	0.0000
HOMO-1	0.0004	0.0003	0.0000	0.0000	0.0001	0.0001
HOMO	0.0004	0.0004	0.0001	0.0001	0.0000	0.0000
LUMO	0.0372	0.0217	0.0213	0.0138	0.0236	0.0172
LUMO+1	0.0073	0.0036	0.0252	0.0186	0.0285	0.0151
LUMO+2	0.0149	0.0085	0.0007	0.0002	0.0010	0.0016
LUMO+3	0.0000	0.0000	0.0001	0.0000	0.0000	0.0001

LUMO+4	0.0001	0.0001	0.0050	0.0039	0.0063	0.0034
LUMO+5	0.0036	0.0020	0.0048	0.0042	0.0064	0.0059
LUMO+6	0.0000	0.0000	0.0000	0.0000	0.0000	0.0000
LUMO+7	0.0000	0.0000	0.0073	0.0081	0.0055	0.0045
LUMO+8	0.0069	0.0060	0.0001	0.0000	0.0003	0.0006
LUMO+9	0.0033	0.0026	0.0005	0.0006	0.0000	0.0000
Total	1.0399	0.8955	0.3803	0.5234	0.5584	0.2957

(e-2)

	NO_2					
	Annulene		Polyene A		Polyene B	
	α orbital	β orbital	α orbital	β orbital	α orbital	β orbital
$d_{1,\sigma}{}^2$	0.224	0.065	0.268	0.104	0.131	0.045
$d_{N,\sigma}{}^2$	0.065	0.224	0.045	0.131	0.104	0.268
$V_{L,\sigma}{}^2$ / eV^2	3.53	3.53	4.27	3.84	3.84	4.27
HOMO-LUMO gap / eV	2.25	2.25	2.55	2.55	2.55	2.55
Fermi energy / eV	-5.09	-5.09	-4.84	-4.84	-4.84	-4.84
$\gamma_{L1,\sigma}$ / eV	0.890	0.481	1.070	0.632	0.710	0.441
$\gamma_{NR,\sigma}$ / eV	0.481	0.890	0.441	0.711	0.632	1.070
$\gamma_{L1,\sigma}{}^2\gamma_{NR,\sigma}{}^2$ / eV^4	0.183	0.183	0.222	0.201	0.201	0.222

Table S3. Orbital energy of (a)NS, (b)OCH_3-substituted, (c)OH-substituted, (d)CN-substituted and (e)NO_2-substituted molecules.

(a)

	NS					
	Orbital energy / eV					
	Annulene		Polyene A		Polyene B	
	α orbital	β orbital	α orbital	β orbital	α orbital	β orbital
HOMO-9	-8.84	-8.84	-8.99	-8.99	-8.99	-8.99
HOMO-8	-8.82	-8.82	-8.98	-8.98	-8.98	-8.98
HOMO-7	-8.82	-8.82	-8.92	-8.92	-8.92	-8.92
HOMO-6	-8.38	-8.38	-8.90	-8.90	-8.90	-8.90

HOMO-5	-7.60	-7.60	-8.71	-8.71	-8.71	-8.71
HOMO-4	-7.59	-7.59	-7.78	-7.78	-7.78	-7.78
HOMO-3	-7.48	-7.48	-7.72	-7.72	-7.72	-7.72
HOMO-2	-7.38	-7.38	-7.54	-7.54	-7.54	-7.54
HOMO-1	-5.93	-5.93	-6.10	-6.10	-6.10	-6.10
HOMO	-5.92	-5.92	-6.02	-6.02	-6.02	-6.02
LUMO	-3.25	-3.25	-3.08	-3.08	-3.08	-3.08
LUMO+1	-2.21	-2.21	-2.30	-2.30	-2.30	-2.30
LUMO+2	-2.15	-2.15	-2.23	-2.23	-2.23	-2.23
LUMO+3	-2.14	-2.14	-2.18	-2.18	-2.18	-2.18
LUMO+4	-1.60	-1.60	-0.49	-0.49	-0.49	-0.49
LUMO+5	-0.11	-0.11	0.28	0.28	0.28	0.28
LUMO+6	0.29	0.29	0.34	0.34	0.34	0.34
LUMO+7	0.36	0.36	0.40	0.40	0.40	0.40
LUMO+8	0.41	0.41	0.57	0.57	0.57	0.57
LUMO+9	0.50	0.50	0.87	0.87	0.87	0.87

(b)

	OCH_3					
	Orbital energy / eV					
	Annulene		Polyene A		Polyene B	
	α orbital	β orbital	α orbital	β orbital	α orbital	β orbital
HOMO-9	-8.83	-8.83	-8.93	-8.93	-8.93	-8.93
HOMO-8	-8.81	-8.81	-8.78	-8.78	-8.78	-8.78
HOMO-7	-8.81	-8.81	-8.71	-8.71	-8.71	-8.71
HOMO-6	-8.37	-8.37	-8.70	-8.70	-8.70	-8.70
HOMO-5	-7.56	-7.56	-8.64	-8.64	-8.64	-8.64
HOMO-4	-7.55	-7.55	-8.00	-8.00	-8.00	-8.00
HOMO-3	-7.04	-7.04	-7.47	-7.47	-7.47	-7.47
HOMO-2	-6.97	-6.97	-7.10	-7.10	-7.10	-7.10
HOMO-1	-5.92	-5.92	-6.30	-6.30	-6.30	-6.30
HOMO	-5.92	-5.92	-5.81	-5.81	-5.81	-5.81

LUMO	-3.13	-3.13	-3.05	-3.05	-3.05	-3.05
LUMO+1	-2.18	-2.18	-2.49	-2.49	-2.49	-2.49
LUMO+2	-2.16	-2.16	-2.17	-2.17	-2.17	-2.17
LUMO+3	-2.14	-2.14	-2.00	-2.00	-2.00	-2.00
LUMO+4	-1.35	-1.35	-0.33	-0.33	-0.33	-0.33
LUMO+5	0.05	0.05	0.08	0.08	0.08	0.08
LUMO+6	0.33	0.33	0.31	0.31	0.31	0.31
LUMO+7	0.34	0.34	0.51	0.51	0.51	0.51
LUMO+8	0.37	0.37	0.62	0.62	0.62	0.62
LUMO+9	0.48	0.48	0.74	0.74	0.74	0.74

(c)

	OH					
	Orbital energy / eV					
	Annulene		Polyene A		Polyene B	
	α orbital	β orbital	α orbital	β orbital	α orbital	β orbital
HOMO-9	-8.89	-8.89	-8.95	-8.95	-8.95	-8.95
HOMO-8	-8.87	-8.87	-8.80	-8.80	-8.80	-8.80
HOMO-7	-8.87	-8.87	-8.74	-8.74	-8.74	-8.74
HOMO-6	-8.47	-8.47	-8.72	-8.72	-8.72	-8.72
HOMO-5	-7.63	-7.63	-8.71	-8.71	-8.71	-8.71
HOMO-4	-7.63	-7.63	-8.06	-8.06	-8.06	-8.06
HOMO-3	-7.16	-7.16	-7.50	-7.50	-7.50	-7.50
HOMO-2	-7.11	-7.11	-7.19	-7.19	-7.19	-7.19
HOMO-1	-5.98	-5.98	-6.35	-6.35	-6.35	-6.35
HOMO	-5.97	-5.97	-5.84	-5.84	-5.84	-5.84
LUMO	-3.21	-3.21	-3.12	-3.12	-3.12	-3.12
LUMO+1	-2.27	-2.27	-2.53	-2.53	-2.53	-2.53
LUMO+2	-2.21	-2.21	-2.22	-2.22	-2.22	-2.22
LUMO+3	-2.20	-2.20	-2.02	-2.02	-2.02	-2.02
LUMO+4	-1.44	-1.44	-0.39	-0.39	-0.39	-0.39
LUMO+5	-0.09	-0.09	0.00	0.00	0.00	0.00

LUMO+6	0.19	0.19	0.30	0.30	0.30	0.30
LUMO+7	0.23	0.23	0.49	0.49	0.49	0.49
LUMO+8	0.35	0.35	0.58	0.58	0.58	0.58
LUMO+9	0.45	0.45	0.73	0.73	0.73	0.73

(d)

	CN					
	Orbital energy / eV					
	Annulene		Polyene A		Polyene B	
	α orbital	β orbital	α orbital	β orbital	α orbital	β orbital
HOMO-9	-9.10	-9.10	-9.10	-9.10	-9.10	-9.10
HOMO-8	-9.08	-9.08	-9.10	-9.10	-9.10	-9.10
HOMO-7	-9.08	-9.08	-9.09	-9.09	-9.09	-9.09
HOMO-6	-8.82	-8.82	-9.09	-9.09	-9.09	-9.09
HOMO-5	-8.04	-8.04	-8.98	-8.98	-8.98	-8.98
HOMO-4	-7.96	-7.96	-7.95	-7.95	-7.95	-7.95
HOMO-3	-7.92	-7.92	-7.93	-7.93	-7.93	-7.93
HOMO-2	-7.83	-7.83	-7.87	-7.87	-7.87	-7.87
HOMO-1	-6.19	-6.19	-6.20	-6.20	-6.20	-6.20
HOMO	-6.18	-6.18	-6.20	-6.20	-6.20	-6.20
LUMO	-3.78	-3.78	-3.43	-3.43	-3.43	-3.43
LUMO+1	-2.76	-2.76	-2.50	-2.50	-2.50	-2.50
LUMO+2	-2.39	-2.39	-2.36	-2.36	-2.36	-2.36
LUMO+3	-2.37	-2.37	-2.35	-2.35	-2.35	-2.35
LUMO+4	-2.35	-2.35	-1.20	-1.20	-1.20	-1.20
LUMO+5	-1.06	-1.06	0.18	0.18	0.18	0.18
LUMO+6	-0.19	-0.19	0.23	0.23	0.23	0.23
LUMO+7	0.17	0.17	0.25	0.25	0.25	0.25
LUMO+8	0.21	0.21	0.38	0.38	0.38	0.38
LUMO+9	0.27	0.27	0.78	0.78	0.78	0.78

(e)

	NO$_2$					
	Orbital energy / eV					
	Annulene		Polyene A		Polyene B	
	α orbital	β orbital	α orbital	β orbital	α orbital	β orbital
HOMO-9	-9.12	-9.12	-9.17	-9.17	-9.17	-9.17
HOMO-8	-9.11	-9.11	-9.09	-9.09	-9.09	-9.09
HOMO-7	-9.11	-9.11	-9.02	-9.02	-9.02	-9.02
HOMO-6	-8.87	-8.87	-9.01	-9.01	-9.01	-9.01
HOMO-5	-8.32	-8.32	-8.97	-8.97	-8.97	-8.97
HOMO-4	-8.07	-8.07	-8.06	-8.06	-8.06	-8.06
HOMO-3	-7.97	-7.97	-7.99	-7.99	-7.99	-7.99
HOMO-2	-7.95	-7.95	-7.85	-7.85	-7.85	-7.85
HOMO-1	-6.22	-6.22	-6.28	-6.28	-6.28	-6.28
HOMO	-6.21	-6.21	-6.12	-6.12	-6.12	-6.12
LUMO	-3.97	-3.97	-3.57	-3.57	-3.57	-3.57
LUMO+1	-3.13	-3.13	-2.54	-2.54	-2.54	-2.54
LUMO+2	-2.61	-2.61	-2.43	-2.43	-2.43	-2.43
LUMO+3	-2.37	-2.37	-2.27	-2.27	-2.27	-2.27
LUMO+4	-2.37	-2.37	-1.79	-1.79	-1.79	-1.79
LUMO+5	-1.99	-1.99	-0.07	-0.07	-0.07	-0.07
LUMO+6	-0.94	-0.94	0.12	0.12	0.12	0.12
LUMO+7	-0.39	-0.39	0.28	0.28	0.28	0.28
LUMO+8	0.06	0.06	0.35	0.35	0.35	0.35
LUMO+9	0.17	0.17	0.55	0.55	0.55	0.55

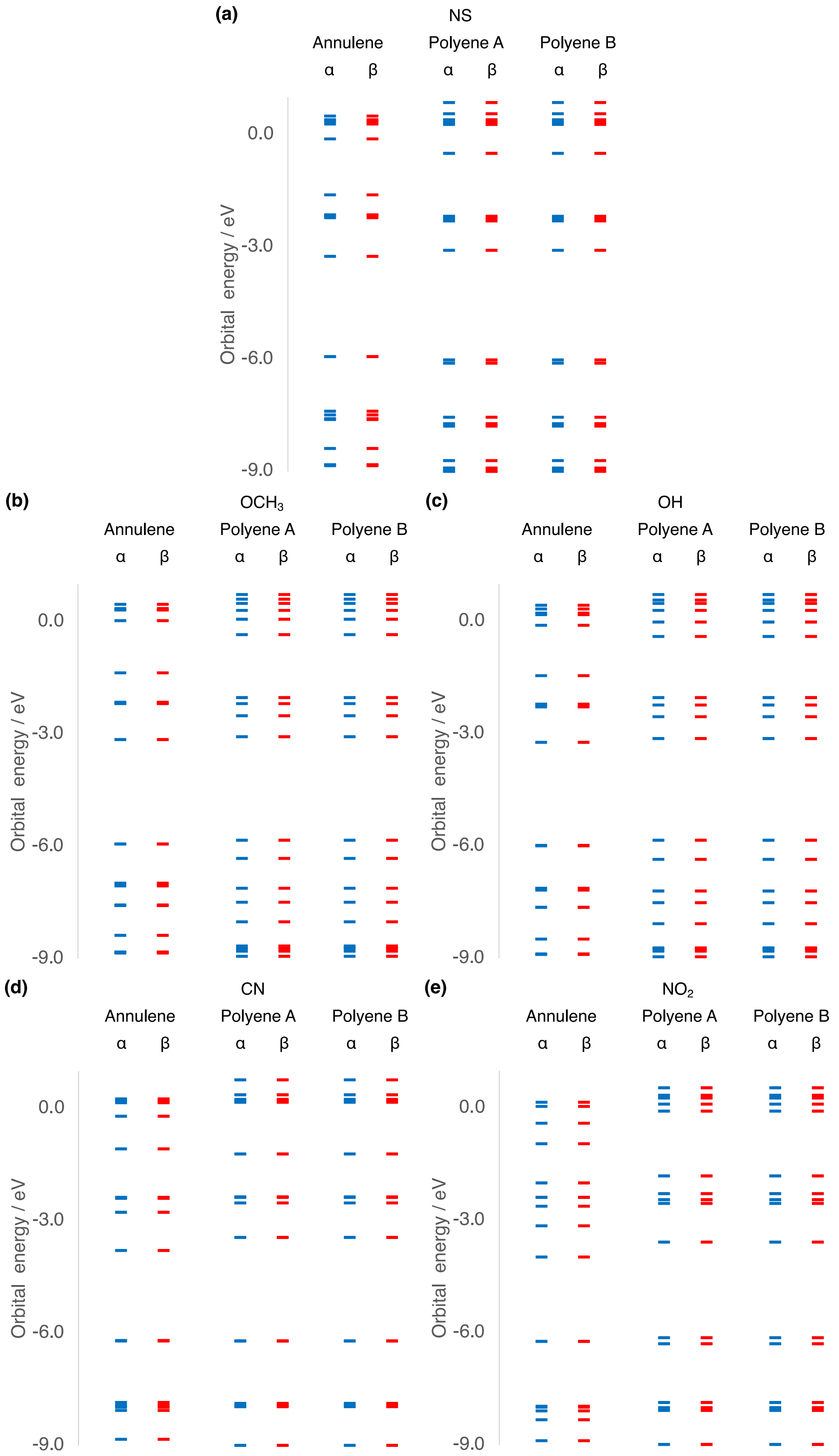

Figure S1. Orbital energy of (a)NS, (b)OCH3-substituted, (c)OH-substituted, (d)CN-substituted and (e)NO2-substituted molecules.

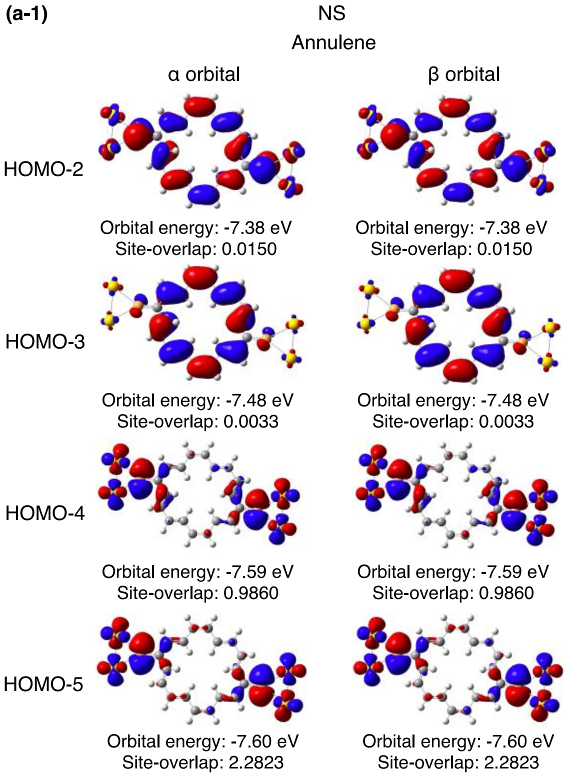
(a-1)
NS
Annulene
α orbital
β orbital
HOMO-2
Orbital energy: -7.38 eV
Site-overlap: 0.0150
Orbital energy: -7.38 eV
Site-overlap: 0.0150
HOMO-3
Orbital energy: -7.48 eV
Site-overlap: 0.0033
Orbital energy: -7.48 eV
Site-overlap: 0.0033
HOMO-4
Orbital energy: -7.59 eV
Site-overlap: 0.9860
Orbital energy: -7.59 eV
Site-overlap: 0.9860
HOMO-5
Orbital energy: -7.60 eV
Site-overlap: 2.2823
Orbital energy: -7.60 eV
Site-overlap: 2.2823

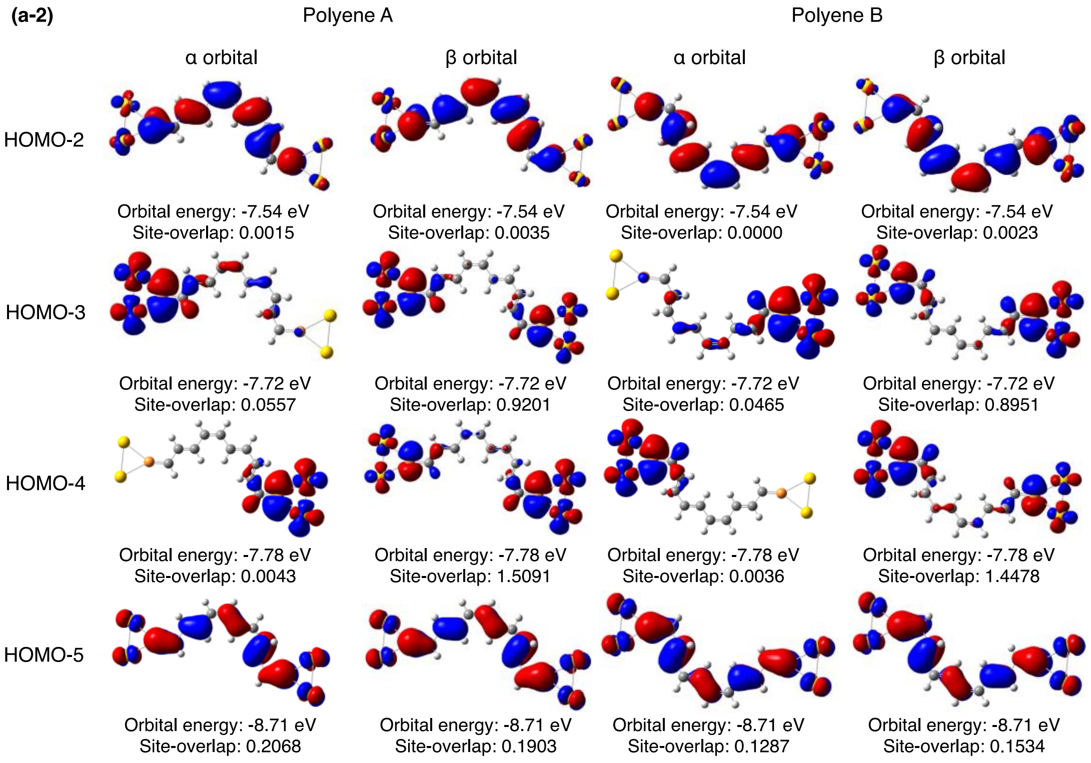
(a-2)
Polyene A
Polyene B
α orbital
β orbital
α orbital
β orbital
HOMO-2
Orbital energy: -7.54 eV
Site-overlap: 0.0015
Orbital energy: -7.54 eV
Site-overlap: 0.0035
Orbital energy: -7.54 eV
Site-overlap: 0.0000
Orbital energy: -7.54 eV
Site-overlap: 0.0023
HOMO-3
Orbital energy: -7.72 eV
Site-overlap: 0.0557
Orbital energy: -7.72 eV
Site-overlap: 0.9201
Orbital energy: -7.72 eV
Site-overlap: 0.0465
Orbital energy: -7.72 eV
Site-overlap: 0.8951
HOMO-4
Orbital energy: -7.78 eV
Site-overlap: 0.0043
Orbital energy: -7.78 eV
Site-overlap: 1.5091
Orbital energy: -7.78 eV
Site-overlap: 0.0036
Orbital energy: -7.78 eV
Site-overlap: 1.4478
HOMO-5
Orbital energy: -8.71 eV
Site-overlap: 0.2068
Orbital energy: -8.71 eV
Site-overlap: 0.1903
Orbital energy: -8.71 eV
Site-overlap: 0.1287
Orbital energy: -8.71 eV
Site-overlap: 0.1534

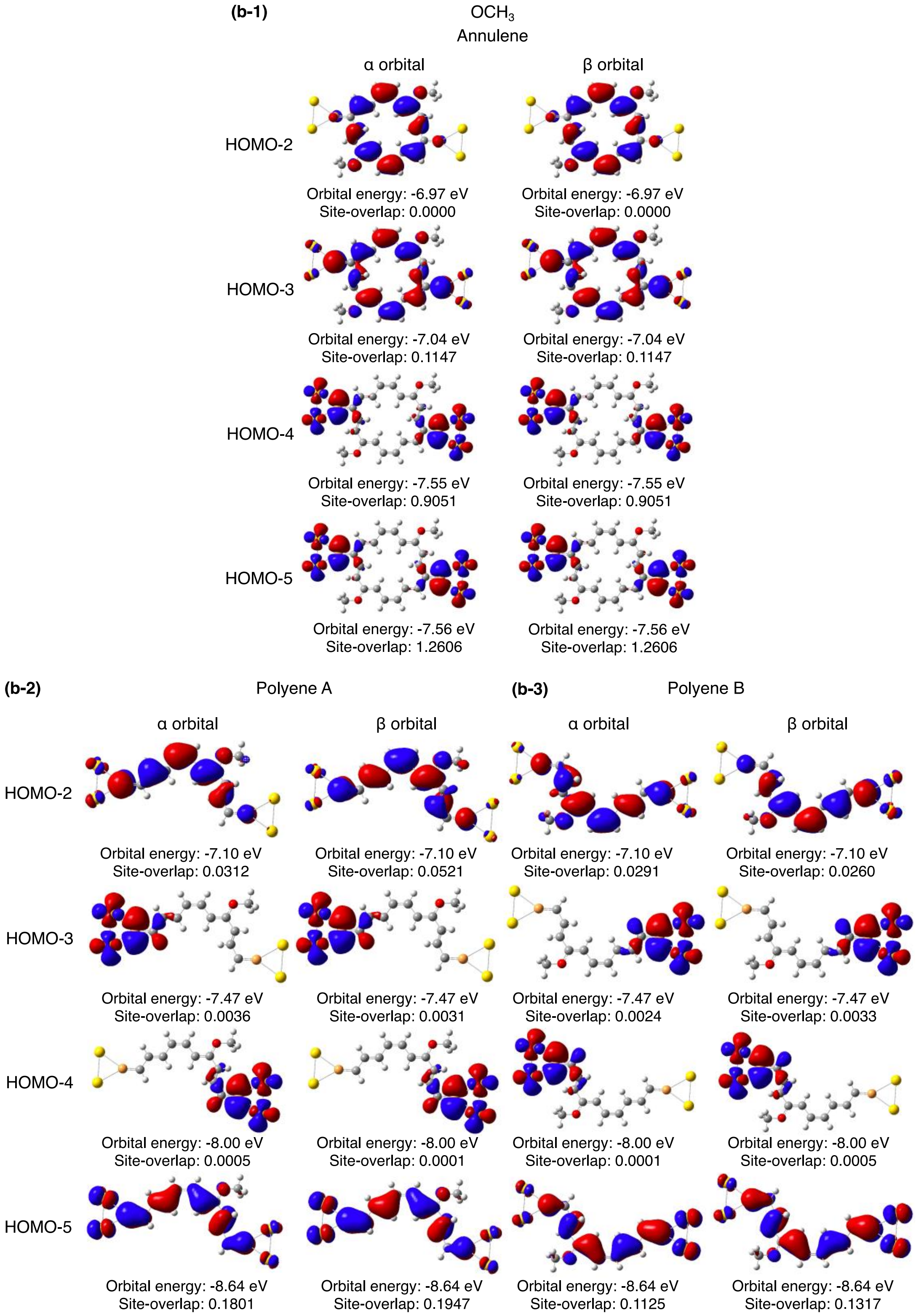
(b-1)
OCH_3
Annulene
α orbital
β orbital
HOMO-2
Orbital energy: -6.97 eV
Site-overlap: 0.0000
Orbital energy: -6.97 eV
Site-overlap: 0.0000
HOMO-3
Orbital energy: -7.04 eV
Site-overlap: 0.1147
Orbital energy: -7.04 eV
Site-overlap: 0.1147
HOMO-4
Orbital energy: -7.55 eV
Site-overlap: 0.9051
Orbital energy: -7.55 eV
Site-overlap: 0.9051
HOMO-5
Orbital energy: -7.56 eV
Site-overlap: 1.2606
Orbital energy: -7.56 eV
Site-overlap: 1.2606
(b-2)
Polyene A
(b-3)
Polyene B
α orbital
β orbital
α orbital
β orbital
HOMO-2
Orbital energy: -7.10 eV
Site-overlap: 0.0312
Orbital energy: -7.10 eV
Site-overlap: 0.0521
Orbital energy: -7.10 eV
Site-overlap: 0.0291
Orbital energy: -7.10 eV
Site-overlap: 0.0260
HOMO-3
Orbital energy: -7.47 eV
Site-overlap: 0.0036
Orbital energy: -7.47 eV
Site-overlap: 0.0031
Orbital energy: -7.47 eV
Site-overlap: 0.0024
Orbital energy: -7.47 eV
Site-overlap: 0.0033
HOMO-4
Orbital energy: -8.00 eV
Site-overlap: 0.0005
Orbital energy: -8.00 eV
Site-overlap: 0.0001
Orbital energy: -8.00 eV
Site-overlap: 0.0001
Orbital energy: -8.00 eV
Site-overlap: 0.0005
HOMO-5
Orbital energy: -8.64 eV
Site-overlap: 0.1801
Orbital energy: -8.64 eV
Site-overlap: 0.1947
Orbital energy: -8.64 eV
Site-overlap: 0.1125
Orbital energy: -8.64 eV
Site-overlap: 0.1317

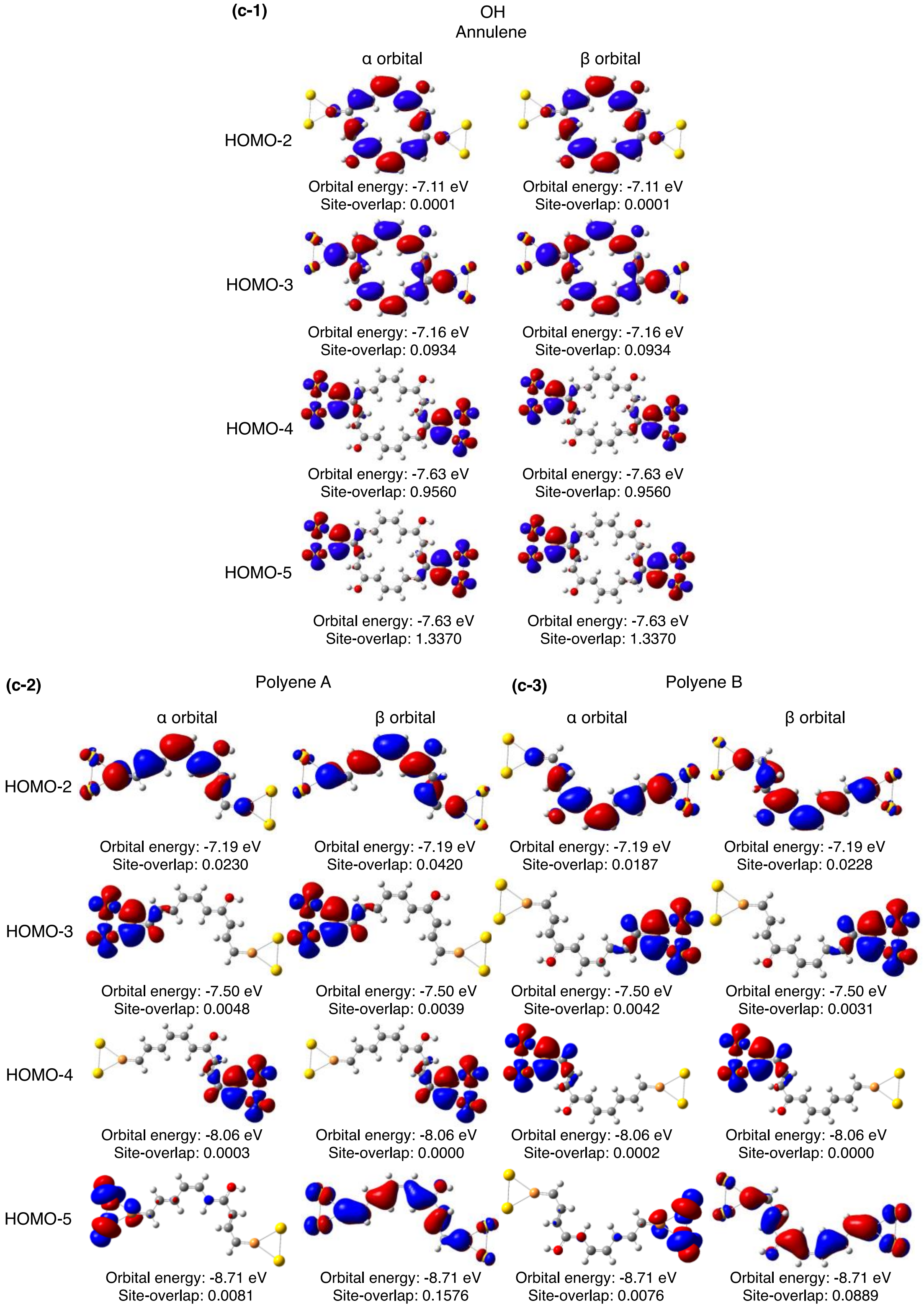

(c-1)
OH
Annulene
α orbital
β orbital
HOMO-2
Orbital energy: -7.11 eV
Site-overlap: 0.0001
Orbital energy: -7.11 eV
Site-overlap: 0.0001
HOMO-3
Orbital energy: -7.16 eV
Site-overlap: 0.0934
Orbital energy: -7.16 eV
Site-overlap: 0.0934
HOMO-4
Orbital energy: -7.63 eV
Site-overlap: 0.9560
Orbital energy: -7.63 eV
Site-overlap: 0.9560
HOMO-5
Orbital energy: -7.63 eV
Site-overlap: 1.3370
Orbital energy: -7.63 eV
Site-overlap: 1.3370
(c-2)
Polyene A
(c-3)
Polyene B
α orbital
β orbital
α orbital
β orbital
HOMO-2
Orbital energy: -7.19 eV
Site-overlap: 0.0230
Orbital energy: -7.19 eV
Site-overlap: 0.0420
Orbital energy: -7.19 eV
Site-overlap: 0.0187
Orbital energy: -7.19 eV
Site-overlap: 0.0228
HOMO-3
Orbital energy: -7.50 eV
Site-overlap: 0.0048
Orbital energy: -7.50 eV
Site-overlap: 0.0039
Orbital energy: -7.50 eV
Site-overlap: 0.0042
Orbital energy: -7.50 eV
Site-overlap: 0.0031
HOMO-4
Orbital energy: -8.06 eV
Site-overlap: 0.0003
Orbital energy: -8.06 eV
Site-overlap: 0.0000
Orbital energy: -8.06 eV
Site-overlap: 0.0002
Orbital energy: -8.06 eV
Site-overlap: 0.0000
HOMO-5
Orbital energy: -8.71 eV
Site-overlap: 0.0081
Orbital energy: -8.71 eV
Site-overlap: 0.1576
Orbital energy: -8.71 eV
Site-overlap: 0.0076
Orbital energy: -8.71 eV
Site-overlap: 0.0889

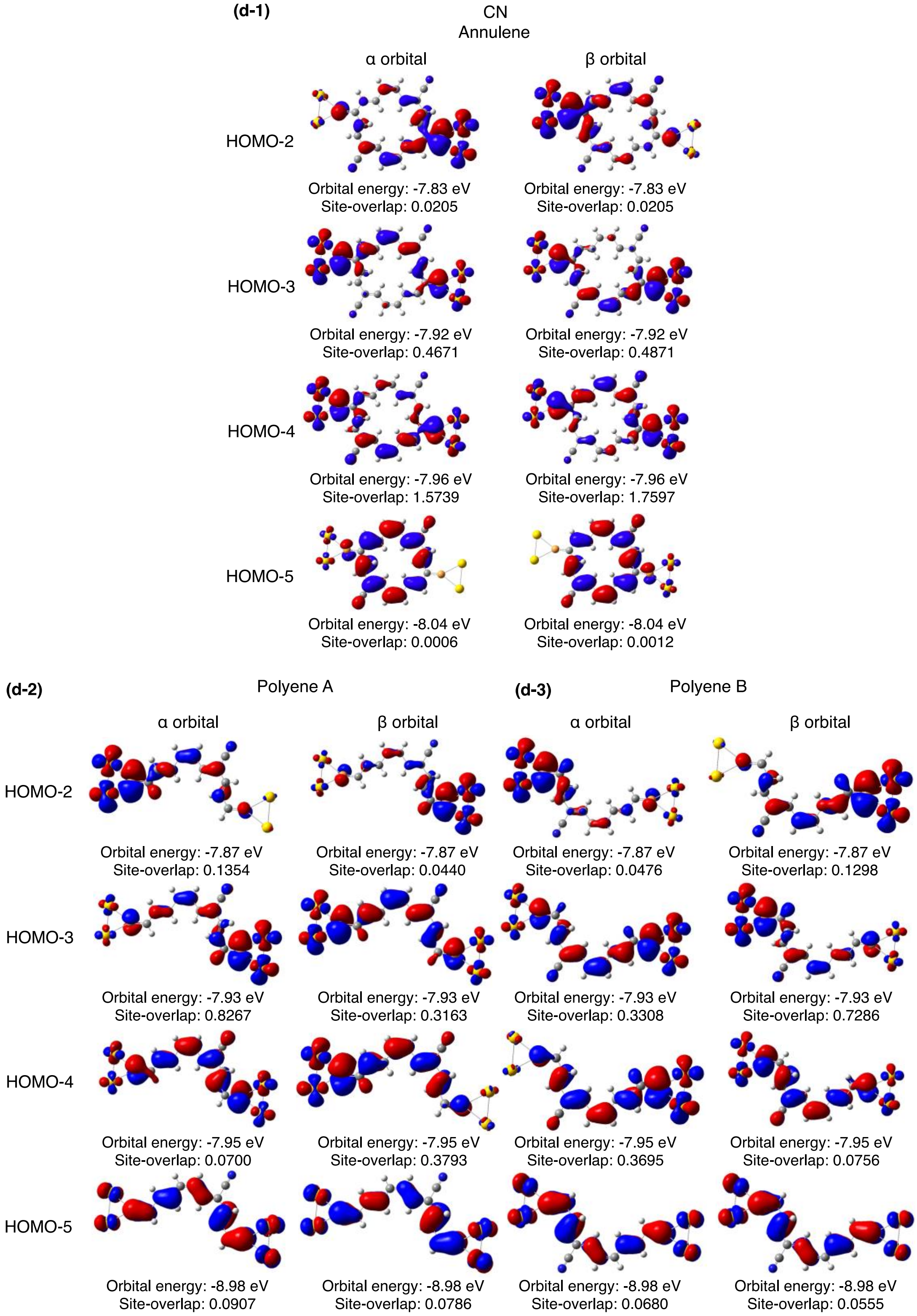
(d-1)
CN
Annulene
α orbital
β orbital
HOMO-2
Orbital energy: -7.83 eV
Site-overlap: 0.0205
Orbital energy: -7.83 eV
Site-overlap: 0.0205
HOMO-3
Orbital energy: -7.92 eV
Site-overlap: 0.4671
Orbital energy: -7.92 eV
Site-overlap: 0.4871
HOMO-4
Orbital energy: -7.96 eV
Site-overlap: 1.5739
Orbital energy: -7.96 eV
Site-overlap: 1.7597
HOMO-5
Orbital energy: -8.04 eV
Site-overlap: 0.0006
Orbital energy: -8.04 eV
Site-overlap: 0.0012
(d-2)
Polyene A
(d-3)
Polyene B
α orbital
β orbital
α orbital
β orbital
HOMO-2
Orbital energy: -7.87 eV
Site-overlap: 0.1354
Orbital energy: -7.87 eV
Site-overlap: 0.0440
Orbital energy: -7.87 eV
Site-overlap: 0.0476
Orbital energy: -7.87 eV
Site-overlap: 0.1298
HOMO-3
Orbital energy: -7.93 eV
Site-overlap: 0.8267
Orbital energy: -7.93 eV
Site-overlap: 0.3163
Orbital energy: -7.93 eV
Site-overlap: 0.3308
Orbital energy: -7.93 eV
Site-overlap: 0.7286
HOMO-4
Orbital energy: -7.95 eV
Site-overlap: 0.0700
Orbital energy: -7.95 eV
Site-overlap: 0.3793
Orbital energy: -7.95 eV
Site-overlap: 0.3695
Orbital energy: -7.95 eV
Site-overlap: 0.0756
HOMO-5
Orbital energy: -8.98 eV
Site-overlap: 0.0907
Orbital energy: -8.98 eV
Site-overlap: 0.0786
Orbital energy: -8.98 eV
Site-overlap: 0.0680
Orbital energy: -8.98 eV
Site-overlap: 0.0555

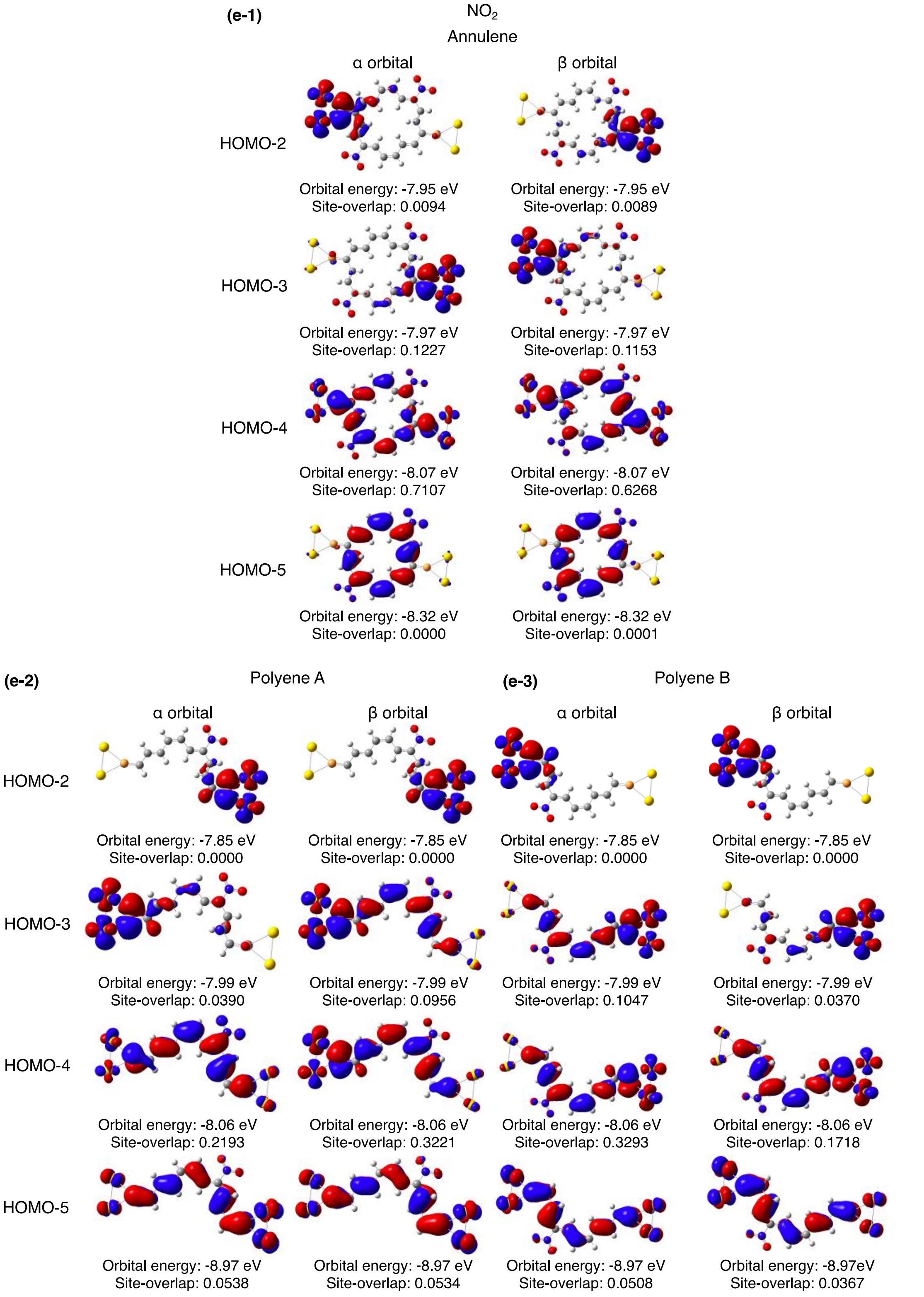

Figure S2. Electron distributions in HOMO-2~HOMO-5 of (a)NS, (b)OCH_3-substituted, (c)OH-substituted, (d)CN-substituted and (e)NO_2-substituted molecules. (x-1)Annulene, (x-2)Polyene A and Polyene B (x: a, b, c, d, e). Their isovalues are 0.02.
